# Multicultural Interactions Decrease the Tendency to View Any Act as Unambiguously Wrong: The Moderating Role of Moral Flexibility

**DOI:** 10.3390/bs15060782

**Published:** 2025-06-05

**Authors:** Liying Jiao, Ying Yang, Yan Xu

**Affiliations:** 1 Department of Psychology, School of Humanities and Social Sciences, Beijing Forestry University, Beijing 100083, China; 2 Beijing Key Laboratory of Applied Experimental Psychology, Faculty of Psychology, Beijing Normal University, Beijing 100875, China; 201721060033@mail.bnu.edu.cn (Y.Y.); xuyan@bnu.edu.cn (Y.X.)

**Keywords:** culture, multicultural experiences, moral judgment, moral flexibility

## Abstract

In four studies, we tested whether individuals’ multicultural experiences influenced their moral judgment. Study 1 found that people’s moral judgments became more lenient after participating in short-term overseas visiting programs using a longitudinal method. Studies 2 and 3 established both correlational and experimental evidence that multicultural interactions (in-depth interactions with multiple cultures)—but not multicultural exposure (superficial exposure to multiple cultures)—predicted less harsh moral judgments. Study 4 explored the psychological mechanism and found that individuals’ moral flexibility moderated the effect of multicultural interactions on moral judgment. Specifically, multicultural interactions reduced the tendency to judge behaviors as unambiguously wrong for individuals with high moral flexibility, while for individuals with low moral flexibility, multicultural interactions did not predict moral judgments. Overall, we found that multicultural interactions readily influenced individuals’ moral judgments, and individuals’ moral character (i.e., moral flexibility) moderated this effect. These results shed light on how moral judgments are influenced by globalization.

## 1. Introduction

Multicultural experiences (MCEs) refer to “exposure to or interactions with elements or members of a different culture(s)” (Maddux et al., 2021, p. 346). In recent decades, the process of globalization in contemporary society has been constantly advancing. Whether active or passive, people in the world are increasingly exposed to different cultures (Leung et al., 2008; J. G. Lu et al., 2017). A growing number of studies has begun to explore the implications of MCEs on individuals’ cognition, behavior, and attitudes (Aytug et al., 2018a; Cheng & Leung, 2012; Maddux & Galinsky, 2009; Tadmor et al., 2012a). Among them, morality is an important outcome of involvement in various cultures in this globalized era. Morality is rooted in and shaped by social culture (Hu, 2017). Studying moral issues from a cultural perspective helps researchers to better understand individuals in different cultures and to reveal the underlying psychological mechanisms of moral diversity (Hu et al., 2018).

In addition to between- and within-cultural differences in moral psychology, researchers have begun to focus on the effects of MCEs on moral judgment, for example, being more lenient toward moral wrongness (Geipel et al., 2015) and making more utilitarian choices (Costa et al., 2014). This research field is still in the preliminary stages, with many questions awaiting further empirical exploration and theoretical construction (Hu et al., 2018). First, prior research on the relationships between MCEs and morality has mostly focused on moral reasoning or immoral behavior (J. G. Lu et al., 2017; Narvaez & Hill, 2010), with less direct attention to moral judgment; this entails an integrated ecological moral cognition and decision-making process possessed by individuals, reflecting their moral norms and moral attitudes, and is one of the core areas of moral psychology (Malle, 2021). Second, MCEs in past research on moral judgments were measured in a fairly homogeneous way (e.g., using a non-native language), and were rarely gauged as a multilevel concept in the context of globalization. Third, the boundary conditions that influence the impact of multicultural experiences on individuals’ moral judgment remain to be explored. In building upon previous research, one important question is how different types of MCEs relate to moral judgments. Another question is how individual factors affect MCEs’ impact on moral judgments. Hence, we attempted to more deeply understand how MCEs influence moral judgments and to examine the individual differences in the MCEs–moral judgments link.

### 1.1. Multicultural Experiences Influence Moral Judgments

MCEs help people integrate seemingly contradictory and incompatible ideas and break down customary fixed systems of knowledge, which may influence individuals’ moral rules (Crisp & Turner, 2011; J. G. Lu et al., 2017). MCEs expose individuals to different moral values and encourage people to learn and integrate different moral rules that can increase their moral relativism (J. G. Lu et al., 2017), which means that moral judgments of right or wrong are not absolute and universal but relative (Goodwin & Darley, 2010; Gowans, 2021; Harman, 1975; Rai & Holyoak, 2013). High moral relativism helps individuals to be more relaxed about moral standards and values (J. Lu et al., 2015; J. G. Lu et al., 2017), to consider more factors of the circumstances that individuals are involved in (Forsyth, 1992; Zaikauskaite et al., 2020), and to make less moral intuitive judgments (Geng et al., 2019), which may result in more lenient immoral or morally controversial behavior (Goodwin & Darley, 2012; Inglehart & Baker, 2000; Kahane et al., 2015; Singh et al., 2007).

In a similar vein, previous studies have investigated the relationship between MCEs and individuals’ moral development. MCE significantly and positively predicted higher post-conventional moral judgment, which implies an increase in the flexibility of moral rules (Endicott et al., 2003; Narvaez & Hill, 2010). In addition, MCEs can influence individuals’ moral choice. For example, using a foreign language can lead to an increase in utilitarian decisions about moral dilemmas (Cipolletti et al., 2016; Costa et al., 2014), and less harsh moral judgments for behaviors that violate social and moral norms (Geipel et al., 2015). In addition, Schema theory states that when individuals encounter new ideas, interact with different people, or try new behaviors, they may encounter a cognitive imbalance, resulting in changes in their response patterns and cognitive schemas (Piaget, 1952). MCEs place individuals in different cultural frameworks and provide them with different moral value systems. These can lead people to consider different contexts or causes when understanding and interpreting moral behaviors, which in turn helps them integrate a more complex and flexible cognitive schema (Endicott et al., 2003; Leung et al., 2008). Thus, individuals with a high number of MCEs are also more likely to consider and understand various situational frameworks when making morally-related decisions, facilitating more lenient moral judgment. Taken together, we hypothesized that MCEs would reduce individuals’ tendency to perceive transgressive acts as unambiguously wrong in moral judgment.

### 1.2. Different Types of MCEs

Not all MCEs will influence moral judgment to the same degree. We therefore maintain that it is valuable to distinguish between two basic types of MCEs: multicultural exposure (ME, e.g., watching foreign movies) and multicultural interaction (MI, e.g., studying in a foreign country). ME describes superficial experiences when a person is exposed to various elements of different cultures without having any interactions with them, while MI refers to profound experiences that include all forms of interactions with individuals or groups in foreign cultures (Aytug et al., 2018a). Although some prior work has begun to distinguish ME and MI in domains such as intergroup bias (e.g., Affinito et al., 2023), limited research has examined how these distinct types of MCEs influence moral judgment. Unlike previous approaches that considered only the breadth and depth of MCEs (e.g., Adam et al., 2018; Cao et al., 2014) or mixed behaviors and attitudes (Endicott et al., 2003), this distinction centers on the experience itself and entails a multidimensional concept of MCE that encompasses the frequency, breadth, and duration of both MI and ME (Aytug et al., 2018a, 2018b).

According to sociocultural theory, human mental functioning is fundamentally a product of culture, and the most important forms of human cognitive activity are developed through interactions within one’s social and physical environment (Tenenberg & Knobelsdorf, 2014). For example, Aytug et al. (2018b) found that creative thinking was enhanced by interactions with (but not exposure to) different cultures. Applying the probability to the moral domain, MCEs’ influence on moral judgment may also be based on MI. On the other hand, from the social cognitive developmental perspective, as a vital aspect of individuals’ cognition, moral judgment is constructed with social interaction as the cornerstone (Young & Heiphetz, 2014). Hence, ME, which means that individuals are exposed to foreign cultures through indirect forms (e.g., books, music) transiently and superficially, may have a lesser effect on individual cognitive growth and values. MI, in contrast, allows individuals to establish relationships with members of other cultures through direct face-to-face communication, making it easier for individuals to form new cognitive schemas. For example, researchers found that both intercultural development and moral judgment were related to the depth of MCEs (Endicott et al., 2003), which are based on interactions. Therefore, we posited that MI (not ME) would have an impact on individuals’ moral judgments.

### 1.3. The Moderating Effect of Moral Flexibility

An individual’s cognition, attitude, and behavior are influenced by both the person and the situation (Allport, 1937; Funder, 2006; Lewin, 1951). Personal factors are also crucial variables to consider in exploring MCEs’ influence on moral judgments. Here, we focused on the role of moral flexibility, which reflects individuals’ flexible moral thinking style and flexible moral systems to adapt to situations (Bartels et al., 2015; Weiss et al., 2018). Compared with individuals that have low flexibility, the cognitive, attitudinal or behavioral outcomes of individuals with high flexibility are more likely to be influenced in the process of experiencing diverse cultures. For example, people who readily use flexible strategies are more likely to accept religious diversity when interacting with different cultures (Gawali & Khattar, 2016). A study conducted among international students showed that the flexibility trait of a multicultural personality could promote people’s greater openness to diversity, which may result in other psychological outcomes, for example, improved psychological adaptation (Yakunina et al., 2012).

The reason why we highlight moral flexibility is that moral judgments are complicated (Bartels et al., 2015), and MCEs influence individuals’ moral behavior and moral decision-making (possibly through a variety of psychological mechanisms, such as accepting the idea that moral rules and principles interact with various cultural elements) (Cao et al., 2014; Tadmor et al., 2012a; J. Lu et al., 2015; J. G. Lu et al., 2017). However, the strength of the influence may be moderated by the individual’s own moral ideology (i.e., whether to emphasize situational factors rather than universal moral rules, Li et al., 2018). For people with high moral flexibility, their moral values and concerns are more likely to be influenced by cultural contexts; thus, MCEs can influence high-level moral flexibility when it comes to individuals’ moral judgments. In contrast, for people with low moral flexibility, their moral decision-making system is rigid and objective; hence, their judgments about what is right or wrong are not easily affected by situational factors. Therefore, contextual factors (i.e., MCEs) might not influence individuals’ moral judgments.

Based on the above reasoning, we developed a research model with all these hypotheses. In general, we proposed that MCEs would decrease the tendency to view an act as unambiguously wrong when making moral judgments. Specifically, MI (other than ME) predicts a more lenient moral judgment. Moreover, individuals’ moral thinking style (i.e., moral flexibility in this study) may moderate MI’s effect on moral judgments. More specifically, MI influences moral judgment only for individuals with high moral flexibility, while for individuals with low moral flexibility, MI may have no effect.

### 1.4. Overview of Studies

The current study aimed to determine whether people’s multicultural experiences influence their moral judgment, and whether this effect varies among individuals with different degrees of moral flexibility. In line with prior research (e.g., Malle, 2021; Ward & King, 2018), we adopt moral judgment as a broad evaluative framework referring to individuals’ evaluative responses to behaviors according to moral standards. A more lenient moral judgment may reflect a reduced tendency to perceive moral transgressions as unambiguously wrong—that is, individuals may be more inclined to consider contextual factors and recognize moral complexity. In Study 1, we employed a longitudinal design to scrutinize college students’ perceived acceptability of several behaviors before and after they studied abroad. In Study 2, we investigated associations between MCEs (MI vs. ME) and the acceptability of numerous behaviors. In Study 3, we examined how MI (vs. ME and control) affect moral judgments about violations by experimental design; this involved a recall task to manipulate the participants’ diverse experiences. Study 4 explored the moderating role of moral flexibility, investigating whether participants’ flexible moral thinking style would moderate MI’s effect on moral judgments.

## 2. Study 1: Studying Abroad Decreases the Tendency to View Acts as Unambiguously Wrong

Study 1 aimed to establish whether people’s moral judgments become more lenient after studying abroad. Specifically, we conducted a two-wave longitudinal study before and after students had completed an international summer exchange program.

### 2.1. Methods

#### 2.1.1. Participants

The participants were students from a university in China who took part in short-term overseas visiting programs during the summer. We performed an analysis using G*Power 3.1 (Faul et al., 2007) to compute the sample size; results showed that 34 participants were needed to have 80% power at an α of 0.05 (two tails), with a medium effect of *d* = 0.50. A total of 48 participants were recruited in the study, and data were taken from 43 participants (10 males, mean age = 21.44 years, *SD* = 2.02) who completed the surveys in both waves. Table 1 provides a detailed report on the demographics of the sample.

#### 2.1.2. Materials

Moral judgments. Moral judgment scenarios are provided in Appendix A. Participants were asked to make judgments of 12 actions that were considered morally wrong (e.g., cheating on a test, tax fraud). These actions were adapted from unambiguously harmful behaviors used in Ward and King’s (2018) study or designed for the current study. The participants rated the acceptability of each behavior on a scale from 1 (*totally unacceptable*) to 10 (*totally acceptable*). The average of all item scores was calculated as a score for moral judgment; for analysis, these ratings were reverse-coded so that higher scores indicate greater unacceptability, thereby reflecting a stronger tendency to view these behaviors as unambiguously wrong. The reliability was α = 0.87 for the pre-test, α = 0.93 for the post-test, and α = 0.71 for the re-test.

#### 2.1.3. Procedure

All students participated in two-week overseas visiting programs organized by the university. The surveys were administered one day before they departed and one day after they returned. The moral judgment questionnaire was measured each time, with items in different orders. Demographic information was collected during the first wave. At each wave, participants were presented with the informed consent form and debriefing statement, and they received CNY 10 (in Chinese yuan, CNY 1 is approximately equal to USD 0.15 when the survey was conducted) as a reward after completing the survey.

### 2.2. Results and Discussion

We performed matched pairs *t*-tests; the results showed that the individuals’ moral judgment at wave 2 (*M* = 8.03, *SD* = 1.66) was significantly lower than at wave 1 (*M* = 8.03, *SD* = 1.77), *t*(42) = −3.35, *p* = 0.002, *d* = −0.51, 95% = [−1.02, −0.25].

Study 1 tested the relationship between MCEs and moral judgment via a longitudinal design, indicating that the participants exhibited a lower tendency to view these behaviors as unambiguously wrong after visiting abroad half a month. However, we did not explore which aspects of MCEs influenced individuals’ moral judgment; it remains unclear whether the impact of being exposed to or interacting with multicultural persons/areas on moral judgments was the same.

## 3. Study 2 Correlational Evidence of MI (Not ME) Affecting Moral Judgments

The main aim of Study 2 was to investigate how individuals’ ME and MI affected their moral judgment. We hypothesized that MI (but not ME) would predict a more lenient moral judgment.

### 3.1. Methods

#### 3.1.1. Participants

We conducted this study mainly to investigate the correlations between MI and moral judgments. We performed a priori power analysis to determine the sample size. Based on Study 1, the effect involved a correlation of ρ H1 = 0.25 and ρ H0 = 0 in the analysis. The power analysis with two-tailed testing revealed a required sample of 123 participants to have adequate power (1 − β = 0.80) at an α of 0.05 (Faul et al., 2007; Kang, 2021).

One hundred fifty-four participants took part in the study, participants who answered incorrectly on the attention check item were excluded. Overall, data from 139 participants (47 males, mean age = 23.58 years, *SD* = 4.93) were included in the analyses.

#### 3.1.2. Materials

Multicultural Experiences. Participants were asked to report their MCEs via the Multicultural Experience Assessment scale (MExA) adapted from a previous study (Aytug et al., 2018a). This scale includes 9 items distinguishing two dimensions of MCEs: ME and MI. The ME dimension, reflecting more passive exposure to other cultures, comprises five items, such as watching movies that take place in different cultures, reading books about foreign people, listening to music of foreign cultures, reading foreign news, and tracking foreign technology and entertainment trends (e.g., celebrity fashion and makeup, tech gadgets). The MI dimension, reflecting direct interpersonal engagement with people from different cultures, includes four items: talking to people from different cultures, socializing with people from different cultures, sharing feelings with people from different cultures, and communicating via writing (e.g., emails, text messages, instant messaging) with people from different cultures. Participants needed to answer each of the 9 items in terms of the following: (1) frequency: the frequency of times that they participated in each activity using the 6-point Likert scale (1 = *never*, 6 = *every day or multiple times a day*), α = 0.86; (2) duration: the approximate number of years that they have been exposed to each activity using a 6-point Likert scale (1 = *not been exposed*, 6 = *exposed for more than 10 years*), α = 0.90; (3) breadth: the approximate number of cultures or countries involved in each activity using a 7-point Likert scale (1 = *not been exposed*; 7 = *exposed more than 5 times*), α = 0.85. The frequency, duration, and breadth scores for the two dimensions were standardized, and averages of these three standardized scores were composited to obtain the multicultural exposure score and the interaction score.

Moral judgments. The materials used to measure moral judgment were the same as those used in Study 1, and the α of the 12 items was 0.84 in the present study.

Demographics. Participants’ gender, age, education (4 categories: ranging from *high school or lower* to *master’s degree or higher*), and annual family income (6 categories: ranging from less than *CNY 50*,*000* to over *CNY 1 million*) were measured in the survey.

Attention check. To ensure that the data were secure, the survey included an attention check item with a particular response option (Curran, 2016): “*Please select the response option slightly disagree.*” We excluded data from participants who answered this item incorrectly in subsequent analyses.

#### 3.1.3. Procedure

The participants were recruited through SoJump, a Chinese online data-collection platform similar to Mturk or Prolific (Dong et al., 2021). All participants were asked to complete the measures online. Each participant was presented with an informed consent form and debriefing statement. They were thanked and paid CNY 5 after completing the survey.

### 3.2. Results and Discussion

#### 3.2.1. Multi-Dimension of MCEs

To examine the underlying structure of multicultural experiences, we conducted confirmatory factor analyses (CFAs) to compare a two-factor model (distinguishing between ME and MI) with a one-factor model (treating all items as indicators of a single latent construct). CFAs were conducted separately for three dimensions of MCEs, frequency, duration, and breadth, using Mplus 8.3 (Muthén & Muthén, 2017). As shown in Table 2, across all three dimensions, the two-factor models demonstrated substantially better fit to the data than the one-factor models. These results support the distinction between ME and MI and suggest that they represent theoretically and empirically separable constructs within each dimension.

#### 3.2.2. MCEs and Moral Judgment

Descriptive statistics and bivariate correlations between each of the two variables are displayed in Table 3. ME and MI were moderately positively correlated, *r* = 0.48, *p* < 0.001. As hypothesized, we found that MI was negatively correlated with moral judgments, *r* = −0.23, *p* = 0.01, and ME was not correlated with moral judgments, *r* = 0.04, *p* = 0.65.

We also conducted regression analysis for moral judgment (see Table 4), and the results showed that when controlling for the demographic variables (Model 1), ME alone did not predict moral judgment (Model 2: β = −0.02, *SE* = 0.09, *p* = 0.81). MI alone significantly and positively predicted moral judgment (Model 3: β = −0.25, *SE* = 0.09, *p* = 0.007). Moreover, the model that included both ME and MI in the regression indicated that only MI could significantly predict moral judgment (Model 4: β_ME→moral judgment_ = 0.09, *SE* = 0.10, *p* = 0.36; β_MI→moral judgment_ = −0.29, *SE* = 0.10, *p* = 0.004), implying that those who experienced more MI rather than ME saw these behaviors as less unambiguously wrong. Additionally, we tested the interaction between ME and MI (Model 5). The interaction effect was not statistically significant (β__ME×MI→moral judgment_ = 0.12, *SE* = 0.09, *p* = 0.18), and only MI significantly predict moral judgment β_ME→moral judgment_ = 0.15, *SE* = 0.11, *p* = 0.18; β_MI→moral judgment_ = −0.33, *SE* = 0.10, *p* = 0.002.

Study 2 provided evidence of a correlation between MCEs and moral judgments. By contrasting the effect of ME versus MI on moral judgments, the results established that MI (i.e., in-depth interactions with multicultural persons/areas) rather than ME (i.e., superficial exposure to multicultural persons/areas) negatively predicted participants’ moral judgments (i.e., the unambiguously wrong judgments of behaviors).

## 4. Study 3: Experimental Evidence of MI (Not ME) Affecting Moral Judgments

Although Study 2 distinguished between the different effects of ME and MI on the influence of moral judgment, it was conducted with a cross-sectional design. Study 3 further explored the causal effects of ME and MI on moral judgment through experimental methods.

### 4.1. Methods

#### 4.1.1. Participants

We carried out a priori power analysis to determine the sample size, referring to Studies 1 and 2 and the results of previous research (J. G. Lu et al., 2017). We expected a medium effect of *f* = 0.25 in the analysis. The power analysis revealed a required sample of 159 participants to have 80% power at an α of 0.05 (Faul et al., 2007). Considering the lower validity of the recall paradigm performed online, we recruited 245 participants to ensure sufficient final valid data.

Only participants who had MCEs were recruited. We excluded participants who answered repeatedly (5 participants), answered the attention check item falsely (17 participants), or did not answer the questions as required (44 participants). The final sample included 179 participants (48 males; *M*_age_ = 23.31 years, *SD* = 3.40).

#### 4.1.2. Materials

MCE manipulation. The manipulation of MCEs refers to previous recall paradigms in MCE studies (Cao et al., 2014; Tadmor et al., 2012a; Tadmor et al., 2018). In the ME condition, participants were asked to recall and write about an impressive experience in which they were indirectly exposed to other cultures (e.g., watching foreign films and TV channels, listening to foreign music, and reading foreign books). In the MI condition, participants were asked to recall and write about an impressive experience in which they were directly interacting with foreigners (e.g., face-to-face/email/SMS communication). In the control condition, participants were asked to recall and write about their last experience of going to a shopping mall or supermarket. Participants in all conditions were asked to describe, in as much detail as possible, their experiences in no less than 200 words.

Manipulation check. As Tadmor et al. (2018) did, we invited a coder who did not know the purpose of the experiment to read every piece of content the participants wrote and coded whether they described one ME, MI, or shopping experience.

Moral judgments. Participants were asked to read four scenarios that described one person committing some moral transgressions (i.e., stealing money, writing a fraudulent resume, evading taxes, or cheating on a recommendation letter) and then rated each one on their agreement based on three dimensions: morally wrong, should be punished, and unacceptable, ranging from 1 (*strongly disagree*) to 7 (*strongly agree*). These scenarios were presented randomly (see Appendix A). The rating scores for all scenarios were combined into moral wrongness, punishment, and unacceptability, and a moral judgment score was composed by averaging these three moral condemnation ratings (α = 0.81), with a higher value reflecting a stronger tendency to view behaviors as unambiguously wrong.

#### 4.1.3. Procedure

Participants were recruited though SoJump and were randomly assigned to one of three conditions: ME, MI, or the control condition. First, they had to perform a recall task after being presented with the online informed consent form. Then, they answered questions about moral judgments and reported their demographic information. After completing all tasks, they were thanked and paid CNY 5.

### 4.2. Results and Discussion

#### 4.2.1. MCEs Manipulation Check

The encoded results showed that all participants accurately wrote about the theme they were assigned; in particular, none of the participants in the control condition wrote about content involving culture or foreign films.

#### 4.2.2. The Effects of ME and MI on Moral Judgments

**Figure 1:**
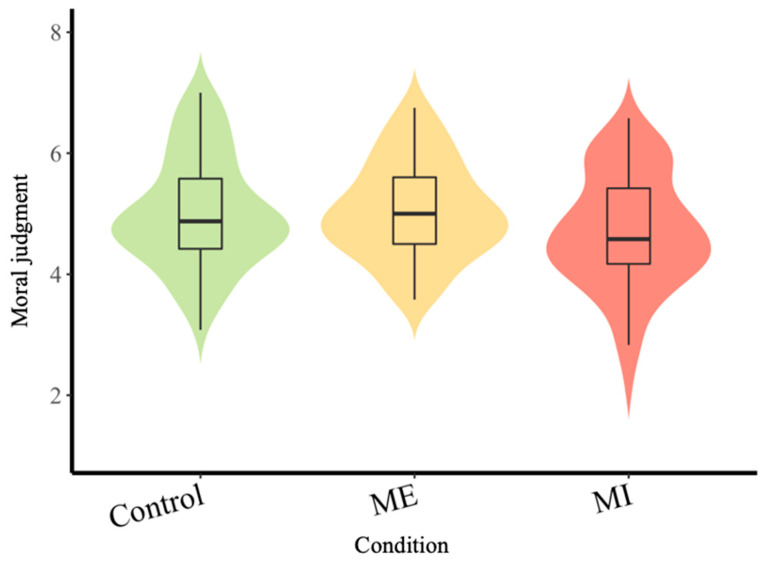
Violin plot with included boxplot of moral judgments in three conditions, Study 3. *Note.* ME = multicultural exposure; MI = multicultural interaction.

We conducted one-way analysis of covariance (ANCOVA) to examine whether there were any significant differences among the three conditions regarding moral judgment when controlling for the demographic variables. The main effect was marginally significant: *F*(2,172) = 2.87, *p* = 0.059, partial η^2^ = 0.03. Then, we conducted post hoc tests to establish differences in moral judgment between any two conditions (see Figure 1). To control the false discovery rate (FDR) for the test, we used Benjamini–Hochberg (BH, Benjamini & Hochberg, 1995)-adjusted *p*-values. The results indicate that participants in the MI condition (*M* = 4.69, *SD* = 0.97) had marginally more lenient moral judgment than those in the ME condition (*M* = 5.05, *SD* = 0.80); the adjusted *p*-value was 0.06, and for those in the control condition (*M* = 5.02, *SD* = 0.93), the adjusted *p*-value was 0.06. Participants in the ME condition were not significantly different from those in the control condition (adjusted *p* = 0.99).

Moreover, we conducted additional analyses to examine whether the differences among the three conditions affected moral wrongness, punishment, and unacceptability. For moral wrongness, there were no differences among the three conditions: *F*(2,172) = 0.29, *p* = 0.75. For punishment, the main effect was significant: *F*(2,172) = 3.53, *p* = 0.03, partial η^2^ = 0.04. The post hoc tests revealed that the participants in the MI condition (*M* = 4.66, *SD* = 1.18) were less likely to punish than those in the control condition (*M* = 5.10, *SD* = 0.96); adjusted *p* = 0.03. There was no significant difference between the MI and ME (*M* = 5.02, *SD* = 0.90) conditions; adjusted *p* = 0.12. For the ME and control conditions, the adjusted *p*-value was 0.39. For the unacceptability, the main effect was significant: *F*(2,172) = 2.88, *p* = 0.025, partial η^2^ = 0.04. The post hoc tests indicated that participants in the MI condition (*M* = 4.08, *SD* = 1.27) were less likely to feel unacceptable than those in the ME condition (*M* = 4.65, *SD* = 1.20), adjusted *p* = 0.04, or in the control condition (*M* = 4.56, *SD* = 1.28), adjusted *p* = 0.04. There was no significant difference between the ME and control conditions (adjusted *p* = 0.87).

For Study 3, we used an experimental design to examine the relationship between MCEs and moral judgment. In line with our hypothesis, the interaction of multi-culture experiences positively predicted lenient moral judgment. The results of the analyses of three indicators of moral judgment showed that while all participants believed these behaviors were morally wrong, individuals in the MI condition considered these behaviors to be less punishable (vs. the control condition) and less unacceptable (vs. the ME and control conditions).

## 5. Study 4: Moral Flexibility Moderates the Effect of MI on Moral Judgments

In Studies 1 to 3, we found that MCEs, especially MIs, decrease the tendency to make unambiguously wrong judgments. However, it remains unclear whether all individuals become less likely to make such rigid moral judgments after experiencing MIs. In Study 4, we tested whether the link between MI and moral judgment was moderated by multicultural attitudes.

### 5.1. Methods

#### 5.1.1. Participants

To determine the sample size, we performed a priori power analysis using G*Power (Faul et al., 2007). The power analysis revealed a required sample of 128 participants for a medium-sized effect of *f* = 0.25, with 0.80 power at an α of 0.05 (two-tailed). Given the ratio of valid samples in Study 3, we recruited 200 participants to ensure final sufficient valid data.

We excluded participants who answered repeatedly (1 participant), answered the attention check item falsely (19 participants), or did not answer the questions as required (23 participants). The final sample included 157 participants (50 males; *M*_age_ = 24.18 years, *SD* = 5.37).

#### 5.1.2. Materials

MCE manipulation. The method of MCE manipulation was the same as in Study 3; participants were asked to recall and write about an impressive experience in which they were directly interacting with foreigners (the MI condition) or shopping for clothes/shoes (the control condition).

Manipulation check. The same as in Study 3.

Moral judgments. The materials used to measure moral judgment were the same as those used in Study 3, and the α of the 12 items was 0.86.

Moral flexibility. Participants completed a 6-item scale used to assess moral flexibility (Shu et al., 2011; Weiss et al., 2018), ranging from 1 (*strongly disagree*) to 7 (*strongly agree*). This scale gauges the degree to which individuals have flexible moral systems to adapt to situations (Bartels et al., 2015), including items such as “*Rules should be flexible enough to be adapted to different situations.*” The average score for all items was calculated as a score for moral flexibility (α = 0.69); a higher score indicates that moral beliefs are more influenced by context.

#### 5.1.3. Procedure

A between-subjects design was conducted in this study; participants were recruited through SoJump and randomly assigned to either the MI or the control condition. After performing a recall task, they were asked to complete a moral judgment task. Next, they reported their moral flexibility, demographics, and certain other questionnaires irrelevant to this study. All participants read the informed consent form and received CNY 5 as a reward after completing the experiment.

### 5.2. Results and Discussion

#### 5.2.1. MCE Manipulation Check

The results showed that all participants accurately wrote about the theme they were assigned, and none of the participants in the control condition wrote about content involving different cultures.

#### 5.2.2. The Effects of MI on Moral Judgments

We conducted one-way ANCOVA to examine whether there were any significant differences between the MI and control condition regarding moral judgment when controlling for the demographic variables. The main effect was non-significant: *F*(1,151) = 1.96, *p* = 0.16, partial η^2^ = 0.01. However, the descriptive statistical results still showed a trend whereby participants in the MI condition (*M* = 4.73, *SD* = 0.95) make less ambiguously wrong judgment than those in the ME condition (*M* = 4.99, *SD* = 0.99).

#### 5.2.3. The Moderating Effect of Moral Flexibility

**Figure 2:**
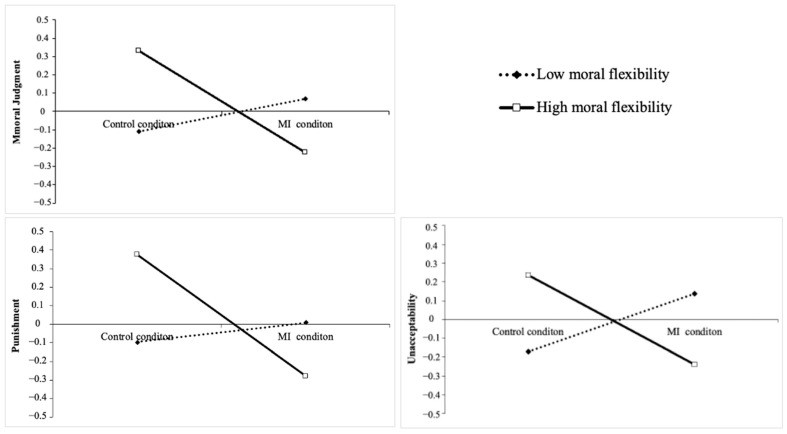
The moderating role of moral flexibility in MI influencing moral judgment (**top** panel), punishment (**below left** panel), and unacceptability (**below right** panel). *Note.* Standardized coefficients are presented. Lower values indicate more lenient moral judgment.

We used Hayes’s PROCESS macro (Hayes, 2018) to examine the moderated model, with control groups encoded as 0, multicultural interaction groups encoded as 1, and moral judgment as the dependent variable, and we controlled for the demographic variables. The results suggest that the interaction between MI and moral flexibility significantly predicted moral judgment: β = −0.37, *SE* = 0.16, *t* = −2.30, 95% CI = [−0.68, −0.05], *p* = 0.02. We further conducted simple slope analysis (Figure 2, upper panel), and the results indicated that for participants with high moral flexibility, MI significantly predicted less harsh moral judgment: β = −0.55, *SE* = 0.21, *t* = −2.59, 95% CI = [−0.98, −0.13], *p* = 0.01. For the participants with low moral flexibility, the relationship between MI and moral judgment was non-significant: β= 0.18, *SE* = 0.23, *t* = 0.78, 95% CI = [−0.27, 0.63], *p* = 0.44.

Subsequently, we conducted additional analyses to examine the moderating effect of moral flexibility on moral wrongness, punishment, and unacceptability. The interaction between MI and moral flexibility did not predict moral wrongness, β = −0.15, *SE* = 0.17, *t* = −0.92, 95% CI = [−0.49, 0.18], *p* = 0.36. However, the interaction between MI and moral flexibility significantly predicted punishment (β = −0.38, *SE* = 0.16, *t* = −2.34, 95% CI = [−0.70, −0.06], *p* = 0.02) and unacceptability (β = −0.39, *SE* = 0.16, *t* = −2.37, 95% CI = [−0.72, −0.07], *p* = 0.02). Simple slope analyses showed that for participants with high moral flexibility, MI significantly and negatively predicted punishment and unacceptability (Figure 2, below panel): β_MI→punishment_ = −0.65, *SE*_MI→punishment_ = 0.22, *t* = −3.00, 95% CI = [−1.09, −0.22], *p* = 0.003; β_MI→unacceptability_ = −0.47, *SE*_MI→unacceptability_ = 0.22, *t* = −2.14, 95% CI = [−0.91, −0.04], *p* = 0.03. For the participants with low moral flexibility, MI did not predict punishment and unacceptability: β_MI→punishment_ = 0.10, *SE*_MI→punishment_ = 0.23, *t* = 0.44, 95% CI = [−0.36, 0.56], *p* = 0.66; β_MI→unacceptability_ = 0.31, *SE*_MI→unacceptability_ = 0.24, *t* = 1.30, 95% CI = [−0.16, 0.77], *p* = 0.20.

Study 4 used an experimental design to examine the relationship between MI and moral judgments and the moderating role of moral flexibility. We did not find a significant effect of MI on moral judgments. However, consistent with expectations, MI negatively predicted moral judgment for individuals with high moral flexibility, whereas the relationship was not significant for individuals with low moral flexibility. Moreover, the results were convergent with Study 3, which showed that the effect of MI on moral judgments occurred only in decision-making about punishments and the unacceptability of behaviors, not in moral wrongness judgments about behaviors.

## 6. Meta-Analytic Overview

To estimate the aggregate effect of MCEs on moral judgment, we performed a meta-analysis of four studies (*N* = 518). The results of Studies 2 and 3 showed that only MI (not ME) influenced individuals’ moral judgment; therefore, we used MI as the independent variable in the meta-analysis. We analyzed the overall moral judgment score in each study as the dependent variable. All of the moral judgment scores were converted into a unified positive direction; a higher score indicates more lenient moral judgment (i.e., less ambiguously wrong judgment).

For Study 1, we calculated Cohen’s *d* between the matched pair samples and then converted Cohen’s *d* into Pearson’s correlation (r) for subsequent analyses. For Studies 2–4, we calculated the correlations (r) between the independent and dependent variables to represent the effect size of each study. Then, we transformed all correlations to Fisher’s z and computed a weighted mean (Goh et al., 2016). The effect was significant, M_r_ = 0.18, Z = 4.03, *p* < 0.001, 95% CI = [0.09, 0.26], suggesting that individuals’ MIs decrease their tendency to view acts as unambiguously wrong.

## 7. General Discussion

The current study found that people who experience multi-cultures have a decreased tendency to make unambiguously wrong judgments via longitudinal, correlational, and experimental designs. Study 1 demonstrates this effect via a longitudinal design. Extending the main finding, Studies 2 and 3 offer evidence that interactions (versus exposure) to multicultural persons/areas predict more lenient moral judgments. Study 4 involved an experimental approach to uncover the moderating role of moral flexibility (i.e., MI prompted more lenient moral judgments for people with high moral flexibility, rather than those with low moral flexibility). These findings extend prior conceptual work distinguishing ME and MI (Aytug et al., 2018b) by providing empirical evidence that MI—but not necessarily ME—is more consistently associated with reduced tendencies to view moral violations as unambiguously wrong. Moreover, by identifying moral flexibility as a key moderator, we contribute to a more nuanced understanding of how multicultural experiences interact with individual dispositions to shape moral judgment.

The results of the present study are in line with previous findings that only MI can predict a higher cognitive outcome (Aytug et al., 2018b). MI influences moral judgments because cognitive growth is socially constructed through interactions (Rogoff, 2003), and when individuals form relationships and interact with members or elements of different cultures, they may easily understand the knowledge and behavioral patterns of different cultures. For example, the development and practice of new schemas are difficult to acquire via books or other inanimate sources; however, they can be acquired through long-term social interactions with individuals or groups in a given culture (Aytug et al., 2018b). Hence, MI contributes to an individual’s cognitive growth through deeper cultural experiences, whereas an ME may have minor effects on schema development because of its transient and superficial nature (Endicott et al., 2003). MCEs influence individuals’ cognition and behavior through deep interactions, which work when individuals have some in-depth understanding and direct involvement with foreign cultures (Maddux & Galinsky, 2009).

We also found a moderating role of moral flexibility in the connection between MI and moral judgments in Study 4. That is, MI affects moral judgments through interactions with the moral flexibility of the person making a judgment. Highly morally flexible individuals are more likely to be affected by environmental factors than rigid ethical principles (Forsyth, 1992; Li et al., 2018); thus, they may be more susceptible to the influence of MCEs when making moral judgments. In fact, person-situation interactions have attracted the attention of social and personality psychologists for decades (Hill & Lapsley, 2009; Zettler & Hilbig, 2010). Thus, the effects of MCEs on individuals’ cognition, attitudes, and behavior may be influenced by person-related variables. For instance, openness to experiences moderates the relationship between MCEs and creativity (X. Chen et al., 2016). Using this perspective in moral domains, individuals’ moral judgment is the product of person-related factors such as personality traits, thinking style, emotion, and ideology (Andrejević et al., 2021; Horberg et al., 2011; Pennycook et al., 2014; Smillie et al., 2021), as well as situational factors such as environmental variability or MCEs (Ding & Savani, 2020; Geipel et al., 2015). Moreover, the results promote further reflection by researchers to explore the psychological mechanisms by which MCEs influence morality.

In addition, the measurement of moral judgment in Studies 3 and 4 adopted a multi-indicator model referring to previous research (Barrett et al., 2016; Ward & King, 2018), which was divided into three indices: moral wrongness, punishment, and unacceptability. The results revealed a separate consequence between moral wrongness and moral punishment/unacceptability (i.e., MI did not predict moral wrongness ratings but did have impacts on judgments of punishment and unacceptability). This is consistent with the wording effect of moral judgments, in which judgments of wrongness and punishment/blame/acceptance about one behavior are different (Barbosa & Jiménez-Leal, 2017; O’Hara et al., 2010). This may be because the participants considered different priorities when making judgments in these dimensions. For example, moral judgments of “right or wrong” rely on the agent’s mental state, whereas judgments of punishment are related to both mental state and the causal connection between the agent and harm outcomes (Cushman, 2008). Overall, MCEs provide an understanding of social practices and conventions in different cultures; to some extent, people’s judgments of punishment and acceptance are more susceptible to MCEs than wrongness.

### Strengths, Limitations, and Future Directions

Globalization has become the primary theme of the era. Political clashes, the economy, trade, and cultural exchanges among countries are constantly advancing (Moghadam, 2021). Multicultural psychology—with its chief feature, which emphasizes the dynamic construction of culture—views culture as a knowledge system that can be activated by initiating external clues (Hou & Zhang, 2012; Chiu & Hong, 2013), focusing on the impact of dynamic and diverse cultural experiences on individuals (S. X. Chen et al., 2016). Integrating the factors associated with the process of globalization and morality can provide a valuable exploratory path for social psychology (Hu et al., 2018; Gelfand et al., 2011). We found that MCEs had an impact on moral judgment, which might prompt people to become concerned about moral development in the current wave of globalization.

Limitations of the current research need to be addressed and point toward possible topics for future research. First, we found only small to moderate effects of MI on moral judgments. The frequency, duration, and breadth of the MCEs in this research were low; moreover, the participants in the longitudinal studied were abroad for a short period (i.e., approximately two weeks); much research has shown than travel abroad is not the same as living abroad and that short experiences do not have lasting effects (e.g., Maddux & Galinsky, 2009). MIs may have different impacts as the frequency, duration, and breadth increase. The impact of richer and deeper MIs on moral judgments may be more noticeable; hence, it remains necessary to examine the conclusions of this research in the context of deeper MCEs. In addition, further research can investigate the thresholds of frequency, duration, and breadth at which the effect of MIs on moral judgments leads to meaningful change (Maddux et al., 2021) and the dynamic shifts toward the impacts of MIs on moral judgment as MIs become deeper.

Second, this study has limitations for generalizability. Specifically, the participants were mostly college students, and their multicultural interaction experiences mostly occurred on campus or in overseas exchange study programs. However, transnational business is becoming increasingly common in the 21st century (Maddux et al., 2021). People might work for foreign companies, foreign countries, or teams with high diversity cultures (Froese et al., 2019; Shipilov et al., 2017; Tadmor et al., 2012b). Future studies can extend the research to more diverse samples to broaden the applicability and impact of new findings.

Third, another limitation is that participants’ moral flexibility in Study 4 was measured in the same period as the experimental condition, and the measurements may have been influenced by MI. To make the conclusion more accurate, future studies should be performed over a longer period, with individual variable measurements and experimental manipulations completed at different times. Fourth, we refined the indicators of moral judgments and found a dissociative effect of MCEs on moral wrongness and punishment/unacceptability. This may be related to the fact that people focus on different points when making “right or wrong” and “punishment” moral judgments (Cushman, 2008), but we did not examine it deeply in this study. Therefore, questions about how MCEs influence different kinds of moral judgments and what the psychological mechanisms are still need to be verified by more empirical studies. Furthermore, our findings revealed differential effects of MEs and MIs. Future research could examine whether these differential effects extend to other moral outcomes.

Additionally, prior research has emphasized that not only the structure of multicultural experiences but also their emotional valence (positive vs. negative appraisal) plays a critical role in shaping individuals’ attitudes and behaviors (Affinito et al., 2023; Barlow et al., 2012; Maddux et al., 2021). While our study primarily focused on the structural distinction of MCEs, future research could further investigate how the valence of these experiences moderates their impact on moral judgment.

Moreover, the set of covariates this study included was limited; there would be other potential alternative explanations that may explain the effect of MI on moral judgment; hence, relevant control variables such as openness to experience and risk taking also need to be taken into account. We also encourage future studies that can control for more key variables to verify the reliability of the research hypothesis.

## 8. Conclusions

The present research provides insights into how MCEs affect individuals’ moral judgments and how individual factors influence this outcome. The results of four studies and one meta-analysis suggest that only MI (not ME) can predict moral judgments, indicating that a high number of MIs decreases the tendency to make unambiguously wrong judgments. Moreover, our findings indicate that individuals’ moral flexibility serves as a moderator between MI and moral judgments; thus, MIs could significantly predict less harsh moral judgment, but only for individuals with high moral flexibility. We recommend that future research explore this effect in the context of deeper MCEs and examine it in different samples for generalizability.

## Figures and Tables

**Table 1:** Sample demographic characteristics in the four studies.

Characteristics		Study 1		Study 2		Study 3		Study 4	
		*n*	%	*n*	%	*n*	%	*n*	%
Gender									
	Male	10	23.26	47	33.81	48	26.82	50	31.85
	Female	33	76.74	92	66.19	131	73.18	107	68.15
Education background									
	High school or lower	-	-	2	1.44	3	1.68	4	2.55
	Junior college	-	-	4	2.88	1	0.56	1	0.64
	Bachelor’s degree	43	100	80	57.55	73	40.78	89	56.69
	Master’s degree or higher	-	-	53	38.13	102	56.98	63	40.13
Monthly income									
	Less than CNY 50,000	5	11.63	30	21.58	36	20.11	24	15.29
	CNY 50,000–CNY 100,000	10	23.26	45	32.37	50	27.93	51	32.48
	CNY 100,000–CNY 200,000	19	44.19	38	27.34	59	32.96	54	34.39
	CNY 200,000–CNY 500,000	8	18.60	21	15.11	29	16.20	23	14.65
	CNY 500,000–CNY 1 million	1	2.33	5	3.60	4	2.23	5	3.18
	Over CNY 1 million	0.00	0.00	0.00	0.00	1	0.56	0.00	0.00
		*M* ± *SD*							
Age		21.44 ± 2.02		23.58 ± 4.93		23.31 ± 3.40		24.18 ± 5.37	

**Table 2:** Model fit indices of the MExA (Study 2).

Entries	Model	χ^2^	*df*	χ^2^/*df*	CFI	TLI	SRMR	RESEA	90% CI for RESEA
Frequency	1-factor	195.71 ***	27	7.25	0.75	0.67	0.15	0.21	[0.19, 0.24]
	2-factor	48.43 **	26	1.86	0.97	0.95	0.05	0.08	[0.04, 0.11]
Duration	1-factor	249.02 ***	27	9.22	0.74	0.65	0.13	0.24	[0.22, 0.27]
	2-factor	89.00 ***	26	3.42	0.93	0.90	0.06	0.13	[0.10, 0.16]
Breadth	1-factor	296.29 ***	27	1.97	0.66	0.54	0.21	0.27	[0.24, 0.30]
	2-factor	55.21 ***	26	2.12	0.96	0.95	0.05	0.09	[0.06, 0.12]

*Note. df*, degrees of freedom. CFI = comparative fit index; TLI = Tucker–Lewis index; SRMR = standardized root mean square residual; RMSEA = root mean square error of approximation. ** *p* < 0.01. *** *p* < 0.001.

**Table 3:** Descriptive statistics and bivariate correlations of the variables (Study 2).

Variables	*M* (*SD*)	α	1	2	3	4	5	6
1. ME ^a^	0.00 (0.83)	0.90	-					
2. MI ^a^	0.00(0.86)	0.94	0.48 ***					
3. Moral judgment	8.89 (0.99)	0.84	−0.04	−0.23 **				
4. Gender	0.34 (0.48)	-	−0.03	0.02	0.13			
5. Age	23.58 (4.93)	-	0.09	0.17 *	−0.04	0.06		
6. Education	3.32 (0.61)	-	0.23 **	0.19 *	0.01	0.05	0.18 *	
7. Income	2.47 (1.10)	-	0.31 ***	0.31 ***	0.10	0.04	−0.04	0.01

*Note.* ME = multicultural exposure; MI = multicultural interaction. Gender: 0 = female, 1 = male. Education: 1 = high school or lower, 2 = junior college, 3 = bachelor’s degree, 4 = master’s degree or higher; Income: 1 = less than CNY 50,000, 2 = CNY 50,000–CNY 100,000, 3 = CNY 100,000–CNY 200,000, 4 = CNY2 00,000–CNY 500,000, 5 = CNY 500,000–CNY 1 million, 6 = over CNY 1 million. ^a^ Standardized values were used to computed these scores. * *p* < 0.05. ** *p* < 0.01. *** *p* < 0.001.

**Table 4:** Regression analysis of moral judgment (Study 2).

Variables	Model 1 β (SE)	Model 2	Model 3	Model 4	Model 5
Constant	0.10 (0.61)	0.05 (0.65)	−0.48 (0.63)	−0.36 (0.65)	−0.37 (0.64)
Control variable					
Gender	−0.27 (0.18)	−0.28 (0.18)	−0.28 (0.18)	−0.27 (0.18)	−0.26 (0.18)
Age	0.01 (0.02)	0.01 (0.02)	0.02 (0.02)	0.02 (0.02)	0.01 (0.02)
Education	−0.01 (0.14)	−0.01 (0.15)	0.05 (0.14)	0.03 (0.14)	0.05 (0.15)
Income	−0.08 (0.08)	−0.07 (0.08)	−0.01 (0.08)	−0.02 (0.08)	−0.03 (0.08)
Key predictors					
ME		−0.02 (0.09)		0.09 (0.10)	0.15 (0.11)
MI			−0.25 (0.09) **	−0.29 (0.10) **	−0.33 (0.10) **
ME × MI					0.12 (0.09)
R^2^	0.03	0.03	0.08	0.09	0.10

*Note.* ME = multicultural exposure; MI = multicultural interaction. Standardized regression coefficients are presented, and standard errors are in parentheses. ** *p* < 0.01.

## Data Availability

The data presented in this study are available on request from the corresponding author.

## Appendix A

Study 1 and 2 Scenarios
  1. Lie on your taxes
  2. Make a fraudulent resume
  3. Take public transportation without buying a ticket
  4. Accept bribes
  5. Cheat on a romantic partner
  6. Cheat on a test
  7. Insult an overweight classmate/colleague
  8. Blame a coworker for your mistake
  9. Appropriate shared bicycles for your own use
  10. Kick a dog
  11. Violence against others
  12. Keep extra change a sales clerk accidentally gives you

Study 3 and 4 Scenarios

Scenario 1

Wang Hua was walking on the street when he found a wallet lying on the ground. Wang Hua opened the wallet and found hundreds of cash and the owner’s driver’s license inside. The credit cards and other objects in the wallet showed that the owner was rich. And Wang Hua’s life has been hit recently, causing economic difficulties, and he really needs some money. Wang Hua decided to secretly leave the cash behind and just return the wallet to the owner.

Scenario 2

Li Feng has been looking for a job recently, but things are not going very well. Li Feng thought that if there was a more impressive resume, the opportunities of being hired would be greatly increased. So Li Feng decided to put some fake information on his resume to make himself stand out more. Li Feng was eventually hired by a company, defeating several candidates who were actually more qualified.

Scenario 3

Zhao Li is a business executive, Zhao’s mother became ill unexpectedly last year and had very high medical expenses. To cover the cost of mother’s surgery, Zhao made false reports of the income, reporting only part of the income while intentionally omitting tens of thousands yuan when paying taxes.

Scenario 4

Zheng Jia is a recent graduate. To increase the opportunities to get a job at a famous company, Zheng wrote a letter of recommendation detailing strengths and being approved by the professor. Zheng Jia signed the professor’s name on the letter without permission (the professor did express approval, but the professor was uninformed of the recommendation letter).
